# Reducing health care-associated infections by implementing separated environmental cleaning management measures by using disposable wipes of four colors

**DOI:** 10.1186/s13756-018-0320-6

**Published:** 2018-03-07

**Authors:** Swee Siang Wong, Cheng Hua Huang, Chiu Chu Yang, Yi Pei Hsieh, Chen Ni Kuo, Yi Ru Chen, Li Ching Chen

**Affiliations:** 1 Department of Internal Medicine, Cathay General Hospital, Taipei, Taiwan; 2 Department of Infection Control, Cathay General Hospital, Taipei, Taiwan; 3 Division of Infection Disease, Cathay General Hospital, Taipei, Taiwan; 4 Division of Medical Intensive Care Unit, Cathay General Hospital, Taipei, Taiwan; 5 Department of Internal Medicine, Division of Infectious Diseases, Cathay General Hospital, No.280, Sec. 4, Ren Ai Rd., Da’an Dist, Taipei City, 106 Taiwan, Republic of China

**Keywords:** Separated environmental cleaning management, Disposable wipes, High-touch surface, ATP bioluminescence test, Healthcare-associated infection

## Abstract

**Background:**

Environmental cleaning is a fundamental principle of infection control in health care settings. We determined whether implementing separated environmental cleaning management measures in MICU reduced the density of HAI.

**Methods:**

We performed a 4-month prospective cohort intervention study between August and December 2013, at the MICU of Cathay General hospital. We arranged a training program for all the cleaning staff regarding separated environmental cleaning management measures by using disposable wipes of four colors to clean the patients’ bedside areas, areas at a high risk of contamination, paperwork areas, and public areas. Fifteen high-touch surfaces were selected for cleanliness evaluation by using the adenosine triphosphate (ATP) bioluminescence test. Then data regarding HAI densities in the MICU were collected during the baseline, intervention, and late periods.

**Results:**

A total of 120 ATP readings were obtained. The total number of clean high-touch surfaces increased from 13% to 53%, whereas that of unclean high-touch surface decreased from 47% to 20%. The densities of HAI were 14.32‰ and 14.90‰ during the baseline and intervention periods, respectively. The HAI density did not decrease after the intervention period, but it decreased to 9.07‰ during the late period.

**Conclusion:**

Implementing separated environmental cleaning management measures by using disposable wipes of four colors effectively improves cleanliness in MICU environments. However, no decrease in HAI density was observed within the study period. Considering that achieving high levels of hand-hygiene adherence is difficult, improving environmental cleaning is a crucial adjunctive measure for reducing the incidence of HAIs.

## Background

Environmental particulate matter may consist of human skin and hair; thus, it serves as a vehicle for transporting dust particles and microbes and enables them to settle near the patients’ bedside areas. This particulate matter plays a crucial role in the transmission of dangerous pathogens, including *Clostridium difficile*, and antibiotic-resistant organisms, such as methicillin-resistant *Staphylococcus aureus* (MRSA), vancomycin-resistant enterococci (VRE), and Carbapenem-resistant *Acinetobacter baumannii*, because health care workers often do not practice appropriate hand hygiene [1–6]. Amy J. Ray et al. demonstrated that contact with contaminated environmental surfaces may have resulted in frequent transfer of VRE onto gloved hands. Contaminated gloves may be a major source of VRE transmission because VRE-colonized patients frequently remain unidentified [1]. Hence, environmental cleanliness is a crucial for reducing contamination burdens.

In recent years, several modalities for assessing environmental cleanliness, such as aerobic colony counts, the use of invisible fluorescent markers that are placed on high-touch room surfaces before cleaning and can be detected using UV-light after cleaning, bioluminescence-based adenosine triphosphate (ATP) technologies, and genomic and polymerase chain reaction-based technologies, have been introduced. However, no consensus exists regarding the benchmark for cleanliness although several studies [7–11] have reported different methods for improving environmental cleanliness for reducing the transmission of nosocomial pathogens. Furthermore, no standard methods are available for assessing the effectiveness of environmental cleaning and disinfection activities. Despite improvements and continuous efforts to optimize isolation practices and increase levels of hand hygiene, the prevalence of infection continues to increase due to the growing resistance of nosocomial pathogens [12].

From 2010 to 2012, in our medical intensive care unit (MICU), the density of health care-associated infection (HAI) increased from 10.4‰ to 17.0‰ (unpublished data). We sought to determine whether environmental cleanliness could be improved by using disposable wipes of four colors to implement separated environmental cleaning management measures. A bioluminescence-based adenosine triphosphate (ATP) modality was used to evaluate the level of cleanliness. This strategy was hypothesized to reduce the density of HAI in the MICU.

## Methods

We performed a 4-month prospective cohort intervention study, between August and December 2013, in the 13-bed MICU of Cathay General hospital, a > 700-bed tertiary care teaching hospital in Taiwan. During the pre-intervention phase, we observed that the MICU’s cleaning staff used traditional reusable wipes soaked with a 0.05%–0.5% sodium hypochlorite solution as a chemical disinfectant for cleaning fixed room surfaces as well as mobile devices, surface of monitors, and bed rails. The isolation room was cleaned last. After completing routine daily environmental cleaning, the wipes were washed with detergent and water to remove the remaining sodium hypochlorite solution, dried, and stored. These wipes were not changed routinely; they were only discarded if they were damaged. Separated environmental cleaning management measures were rarely implemented because the sequence in which surfaces were to be cleaned was difficult to memorize. The cleaning staff could not remember exactly which wipes had been used to perform terminal cleaning in empty rooms; the waste room; the isolation room, which contains multiple-drug-resistant organisms; or the nursing station. A visual inspection method was used for evaluating the cleanliness of the wiped surfaces. This method only indicated whether foreign material (e.g. dust and soil) and organic material (e.g. blood, secretions, excreta, and microorganisms) were visible.

After 1 month of the pre-intervention phase, we conducted a training program for all the cleaning staff and related personnel regarding the implementation of separation management measures during routine daily environmental cleaning and terminal cleaning by using disposable wipes of four different colors to clean patient’s bedside areas, high-risk contamination areas, paperwork areas, and public areas. Red wipes were used to clean areas at a high risk of contamination, namely toilets, isolation rooms, and waste rooms; these wipes were discarded daily. Yellow wipes were used to clean the patients’ bedside areas and were discarded after every terminal cleaning. Green wipes were used to clean paperwork areas such as the nursing station and meeting room. Blue wipes were used to clean public areas. The blue and green wipes were discarded every 3 days. Fifteen high-touch surfaces (toilet doorknobs, bedrails, bedside tables, bedside electrocardiogram monitors, bedside flow meters, nursing stations, meeting rooms, procedure tables, telephones, refrigerator handles, and water dispenser buttons) were selected to conduct the ATP bioluminescence test for evaluating their cleanliness. The 15 high-touch surfaces were sampled before and after cleaning. The efficiency of cleaning of the 15 high-touch surfaces was evaluated by using ATP bioluminescence test results. The cleaning staff was informed in advance regarding when ATP measurements would be made before cleaning and after cleaning. An ATP bioluminescence value < 100 was defined as pass (clean) and a value > 250 was defined as fail (unclean), 101–250 was defined as caution (needs enhanced cleaning).

Data on the HAI density of the MICU were collected during the three periods, namely the baseline (May–August 2013), intervention (September–December 2013), and late (January–April 2014) periods Others variables including admissions to MICU, patient-day, Acute Physiology and Chronic Health Evaluation II score, monthly occupancy of the MICU, and length of MICU stay were also measured. The cleaning staff continued to implement separated environmental cleaning management methods by using different colored wipes for routine environmental cleaning and terminal cleaning, and the staff was informed in advance when no ATP measurements would be made before and after cleaning at the late period. The hospital’s Institutional Review Board approved the study protocol.

### Statistical analysis

Categorical variables were analyzed using the χ^2^ statistic and one-way analysis of variance. A *p* value < 0.05 was considered statistically significant.

## Result

**Fig. 1:**
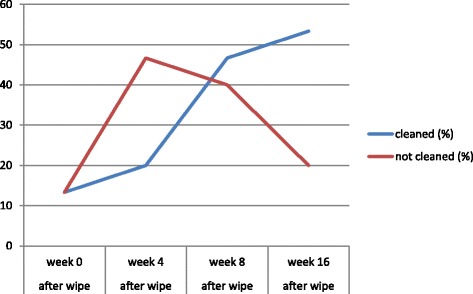
Percentage of total high-touch surfaces cleaned

A total of 120 ATP readings were obtained before and after cleaning in the MICU. The ATP readings were expressed as relative light units. The results revealed that meeting rooms and procedure tables were not significantly cleaner after cleaning than before cleaning (Table 1). The median number of relative light units was significantly lower (i.e. surfaces were cleaner) after cleaning than before cleaning for all 15 high-touch surfaces (Table 1). Within the study period, the total number of high-touch surfaces that were clean increased from 13% to 53%, whereas the total number of high-touch surfaces that were unclean decreased from 47% to 20% (Fig. 1). The results showed that the use of disposable wipes of different colors as well as that of the correct wiping methods or wiping processes could improve environmental cleanliness.

**Table 1:**
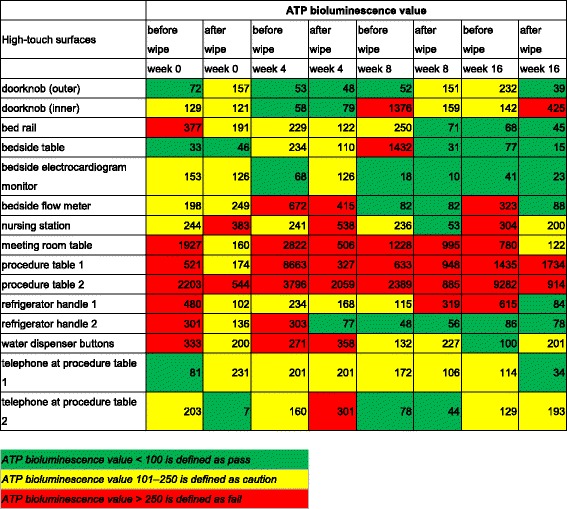
ATP bioluminescence in relative light units

A total of 635 admissions to the MICU were studied. Mean monthly occupancies in the MICU ranged from 96% to 98.8% patients per month. The mean duration of stay ranged from 6.1 to 8.4 days. The densities of HAI during the baseline (May–August 2013), intervention (September–December 2014), and late (January–April 2014) periods were 14.32‰, 14.90‰, and 9.07‰, respectively. No significant differences (*p* = 0.33) were observed among the HAI densities during the three periods (Table 2).

**Table 2:** Acquisition of HAI after enforcement of routine environmental cleaning measures

Variables	baseline period (May–August, 2013)	intervention period (September–December, 2013)	late period (January– April, 2014)	*p* value
Admissions to MICU (number)	200	250	185	
patient-days	1536	1544	1544	0.972
Duration of MICU stay (mean days)	7.7	6.1	8.4	0.02
Monthly occupancy in MICU (%)	96	97.4	98.8	0.147
APACHE II score, mean	15.4	15.8	16.8	0.278
Patients with HAI	22	23	14	0.51
UTI	6	7	2	0.391
Pneumonia	2	2	4	0.418
SSI	1	0	0	N/A
BSI	9	12	7	0.875
others	4	2	1	0.329
Infection density, ‰	14.32	14.90	9.07	0.327

*UTI* Urinary tract infection, *PNEU* Pneumonia, *SSI* Surgical site infection, *BSI* Blood stream infection, *APACHE II* Acute Physiology and Chronic Health Evaluation II

## Discussion

To our knowledge, nosocomial pathogens may be acquired exogenously or may be present among the indigenous microflora of patients at admission [13–16]. Small numbers of these pathogens are either intermittently ingested or released into the environment by the hands of health care workers, who exhibit low levels of compliance with recommended hand hygiene practices. Donskey suggests that reducing the burden of pathogens present on patients’ skin and on environmental surfaces might potentially reduce transmission by reducing the number of microorganisms transferred to the hands of health care workers, thus reducing the transmission of pathogens from environmental surfaces to the patients’ skin [12].

Several studies [5–10, 17] have found that reducing environmental contamination may facilitate the control of the spread of some antibiotic-resistant bacteria in hospitals. The Centers for Disease Control and Prevention and the Healthcare Infection Control Practices Advisory Committee have made numerous recommendations for environmental infection control in health care facilities [18]. Several studies [7, 8, 10] have reported the use of educational interventions directed at cleaning staff. Moreover, neither cultured VRE nor *C. difficiles* was used as a marker organism to evaluate the effectiveness of cleaning and disinfecting activities. Invisible fluorescent markers have also been used previously to monitor cleaning practices. All these strategies can result in improved decontamination of environmental surfaces. However, no consensus exists regarding the benchmarks for cleanliness; comparisons of application methods have not been reported; and contamination assessment techniques are unknown.

The cost and logistical complexity of studying cultures from heavily used environments have precluded health care facilities from using methods for continuously monitoring routine environmental cleanliness. Our study emphasizes that an educational intervention for the cleaning staff regarding the use of disposable wipes of different colors. Educational intervention can facilitate effective implementation of separated environmental cleaning management measures that may result in improved decontamination of environmental surfaces. We used a bioluminescence-based ATP method to detect the presence of organic matter on surfaces before cleaning and after cleaning. ATP testing can be used to provide immediate feedback on surface cleanliness, thus revealing the deficiencies in cleaning protocols and techniques used by the cleaning staff [19–21].

Effectiveness cleanliness was not observed at the nursing station (Tables 1 and 2). This may be because the health care workers had the highest frequency of contact with these surfaces. The total number of clean high-touch surfaces increased at the end of the study period, thus showing that the cleaning staff implemented separated environmental cleaning management measures by using disposable wipes of different colors; this may have provided better environmental cleanliness than that obtained with reusable wipes.

However, we did not observe a decrease in the HAI density during the intervention period. Hence, only 50% of the total number of high-touch surfaces being clean is not sufficient for reducing the bioburden on health care workers’ hands, which transmit microorganisms from environmental surfaces to the patients’ skin, particularly if the workers exhibit low adherence to hand hygiene protocols. Moreover, the educational intervention period (4 months) may not have been sufficiently long to improve environmental cleanliness significantly. Our study documented a widespread deficiency in a fundamental aspect of infection prevention. We must use an objective cleanliness monitoring system, conduct educational and administrative interventions for cleaning staff, and provide continuous performance feedback to the cleaning staff to improve environmental cleanliness.

Our study has several limitations. This study is based on data from a single unit in a single hospital. We did not culture multidrug resistant organisms, VRE, MRSA, or *C. difficiles* before cleaning and after cleaning as marker organisms to evaluate the effectiveness of cleaning and disinfecting activities. Determining the significance of environmental contamination and proving its involvement in cross-acquisition of HAI in health care settings is difficult. In addition, we did not evaluate the effects of enhancement of hand hygiene adherence and compliance of health care workers because these factors may play a crucial role in reducing HAI; the selection of these contributors limits the generalization of our findings.

Although we know that improved compliance of hand hygiene may be the most effective means to reduce HAI density, [22–29] the success of hand hygiene improvement requires the cooperation of all health care workers; however, achieving high levels of cooperation in situations with high workloads and high a demand for disinfection, such as intensive care units, is difficult. Hence, improving environmental cleaning is a crucial adjunctive measure to reduce densities of HAI [8].

## Conclusion

Implementing separated environmental cleaning management measures by using disposable wipes of four colors effectively improves cleanliness in MICU environments. However, no decrease in HAI density was observed within the study period. Considering that achieving high levels of hand-hygiene adherence is difficult, improving environmental cleaning is a crucial adjunctive measure for reducing the incidence of HAIs.
